# Routine Lymph Node Dissection in the Surgical Treatment of Primary Liver Tumors: a Systematic Review and Meta-Analysis

**DOI:** 10.1007/s12029-026-01516-9

**Published:** 2026-07-16

**Authors:** Britte L. Buisman, Lucas Goense, Frederik J. H. Hoogwater, Maarten W. Nijkamp, Inne H. M. Borel Rinkes, Jeroen Hagendoorn, Jelmer E. Oor

**Affiliations:** 1 Department of Surgical Oncology, University Medical Centre Utrecht, Heidelberglaan 100, Utrecht, 3584 CX the Netherlands; 2 Department of Hepato-Pancreato-Biliary Surgery and Liver Transplantation, University Medical Centre Groningen, Groningen, the Netherlands

**Keywords:** Hepatocellular Carcinoma, Intrahepatic Cholangiocarcinoma, Hilar Cholangiocarcinoma, Lymphadenectomy, Lymph node metastasis

## Abstract

**Purpose:**

The incidence of LNM and survival outcomes following routine lymph node dissection (LND) in patients undergoing resection of hepatocellular carcinoma (HCC), intrahepatic- (ICC) and perihilar cholangiocarcinoma (PCC) has not been well-defined. This systematic review and meta-analysis analyzes the incidence of LNM and survival outcomes following routine LND independently of pre-or intraoperative suspicion of LNM following resection of HCC, ICC, and PCC.

**Methods:**

The PubMed database was searched from database inception until March, 2025. Random-effects meta-analysis of the incidence of LNM and hazard ratios (HRs) of overall survival (OS) were conducted for each malignancy.

**Results:**

Forty studies were included. Routine LND was performed in 5 872 patients. The pooled incidence of LNM following routine LND was 5% among 601 HCC patients, 37% among 2 565 ICC patients, and 39% among 2 706 PCC patients. Presence of LNM was associated with worse OS in ICC (HR: 2.46, 95% CI 1.66–3.65) and PCC (HR: 2.01, 95% CI 1.74–2.33) patients.

**Conclusion:**

Routine LND should be performed during resection of ICC and PCC due to the high incidence of LNM and improved survival outcomes, but not during resection of HCC. The presence of LNM gives inferior survival outcomes in ICC and PCC patients.

**Supplementary Information:**

The online version contains supplementary material available at 10.1007/s12029-026-01516-9.

## Introduction

Liver malignancies are the fourth leading cause of cancer mortality worldwide [1]. The most common primary liver malignancy is hepatocellular carcinoma (HCC), representing 75–80% of cases, followed by cholangiocarcinoma (CCA), representing 10–15% of cases [2]. Cholangiocarcinoma includes intrahepatic (ICC)- and perihilar cholangiocarcinoma (PCC). The rising incidence of primary liver malignancies is attributable to improved detection and increased prevalence of risk factors, including liver cirrhosis, metabolic dysfunction-associated steatotic liver disease (MASLD), and smoking [2]. 

Although CCA and HCC are two different clinicopathologically entities, surgical resection with microscopically negative margins remains the optimal treatment for patients with HCC, ICC and PCC [2]. These primary liver malignancies are associated with a high risk of recurrence and poor survival [2, 3]. Less than half of the patients are eligible for curative resection due to locally advanced or metastatic disease and the 5-year overall survival (OS) rates following resection range between 12 and 70% for HCC [3–5], 20–40% for ICC [3, 4], and 20–47% for PCC [6]. The presence of LNM is common in patients with primary liver malignancies and associated with an inferior prognosis [3–7]. 

Recent guidelines recommend routine LND during resection of ICC and PCC to adequately stage patients [2, 8]. However, significant variability exists in the performance and extent of LND between and within countries [3, 4, 9–11]. In fact, LND is still frequently being performed based on pre- or intraoperative suspicion of LNM [4, 9, 10]. Previous systematic reviews and meta-analyses reported a high incidence of LNM and found no survival benefits of LND during resection of HCC, ICC, and PCC [4, 9, 10, 12–15]. However, these meta-analyses were limited by the inclusion of studies that performed selective LND based on pre- or intraoperative findings, overlapping cohorts, and variability in the extent of LND. In contrast, recent studies suggest that routine LND being performed independently of pre- or intraoperative suspicion of LNM during resection of ICC improves survival outcomes and reduces the risk of tumor recurrence without increasing complications [16–18]. 

The objective of this systematic review and meta-analysis is to provide a systematic and the most extensive overview of studies reporting on outcome of routine LND in patients undergoing resection for primary liver cancers, and specifically to analyze the pooled incidence of LNM and survival outcomes in patients following routine LND independently of pre- or intraoperative suspicion of lymph node metastases.

## Materials and methods

### Search Strategy

Following the Preferred Reporting Items for Systematic Reviews and Meta-analyses (PRISMA) guidelines, the PubMed database was systematically searched from database inception until October 13, 2025 [19]. The search terms included ‘hepatocellular carcinoma,’ ‘cholangiocarcinoma,’ ‘lymphatic metastasis,’ and ‘lymphadenectomy.’ The full search syntax is provided upon request. No search filters were applied.

### Study Selection

Articles were screened by title and abstract. Relevant studies were selected for full-text review. Randomized controlled trials (RCTs) and prospective and retrospective studies with at least 20 patients undergoing routine LND independently of pre- or intraoperative suspicion of LNM during resection of HCC, ICC and PCC were considered for inclusion. Studies had to report the number of patients receiving routine LND and the incidence of LNM on histological examination. When studies were published by the same author or included data from overlapping cohorts, the largest or most recent study was selected. Studies were excluded that performed LND based on pre- or intraoperative findings or reported on fibrolamellar HCC, mixed HCC-ICC, and distal cholangiocarcinoma. Non-English studies, systematic and literature reviews, studies without available abstract or full text, expert opinions, letters to the editors, and case reports or series were excluded. Two authors (B.L.B., J.E.O.) reviewed the study selection.

### Data Extraction

Extracted data included: (1) study characteristics: first author, year of publication, country, study design; (2) patient characteristics: total number of patients, number of patients with and without routine LND; (3) oncological characteristics: tumor size, incidence of LNM, most frequent LNM region; (4) treatment characteristics: extent of LND, number of retrieved lymph nodes, rate of resections with microscopically negative (R0) margins; (5) median disease-free survival (DFS) and overall-survival (OS), and 1- and 5-year DFS and OS. In data involving Inverse Probability of Treatment Weight (IPTW) adjustment, data before adjustment were used.

### Outcomes of Interest

The primary outcome was the pooled incidence of LNM on histological examination following routine LND in patients undergoing resection of HCC, ICC and PCC. Secondary outcomes were DFS and OS in patients with (LND+) and without (LND-) routine LND, and in those with (LNM+) and without (LNM–) the presence of LNM.

### Quality Assessment

The risk of bias in RCTs was evaluated with the risk-of-bias (RoB-2) tool and in nonrandomized studies with the risk of bias in non-randomized studies- of interventions (ROBINS-I) tool [20, 21]. The RoB-2 tool assessed the risk of bias as ‘low,’ ‘high’ or ‘with some concerns’ across five domains. The ROBINS-I tool assessed the risk of bias as ‘low,’ ‘moderate,’ ‘serious,’ ‘critical’ or ‘no information’ across seven domains. Studies with critical risk of bias were excluded.

### Statistical Analysis

Meta-analysis of the incidence of LNM and hazard ratios (HRs) of OS were performed for each malignancy by one author (L.G). The proportion of LNM and its 95% confidence interval (CI) were pooled and presented in forest plots. Hazard ratios with 95% CIs of OS comparing LNM + and LNM- patients were extracted and pooled. Meta-analysis was not conducted if fewer than three studies reported an outcome measure. When studies reported outcomes from univariable and multivariable analyses, the univariable outcomes were used. If only Kaplan-Meier curves were provided, HRs and 95% CIs were estimated using WebPlotDigitizer 4.6 as proposed by Tierney et al. [22] Meta-analysis was conducted with logit transformation and a random-effects model. Heterogeneity was assessed using the *I*^*2*^ test (< 30%, mild; 30–50%, moderate; >50%, substantial). Between-study heterogeneity was explored using random-effects meta-regression with study factors including geographic region, year of publication, risk of bias, study sample size, and hazard ratios reconstructed from published Kaplan-Meier curves. Publication bias was assessed with funnel plots and Egger’s regression test for each pooled outcome with at least 10 included studies. Leave-one-out sensitivity analyses were performed for all four pooled outcomes to assess the influence of individual studies on the pooled estimate. Statistical significance was defined as a p-value < 0.05. Meta-analyses were conducted in R software using ‘Rcurl’, ‘metafor’, and ‘meta’ packages (http://www.R-project.org).

## Results

### Study Selection

**Fig. 1:**
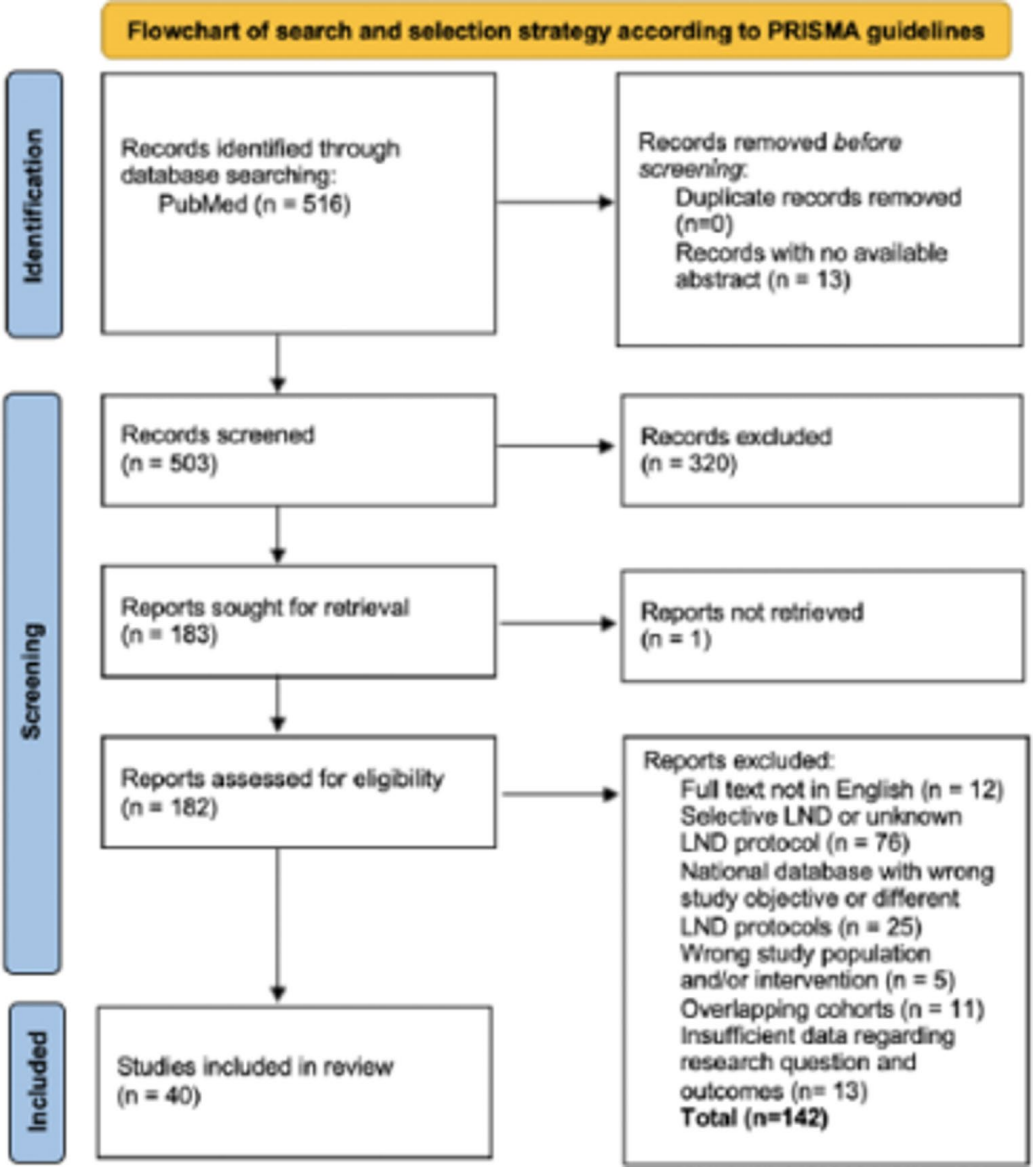
P RISMA flow diagram of search strategy and study selection

The search identified a total of 516 articles (Fig. 1). After title and abstract screening, 183 articles were selected for full-text review. Forty studies published between 1998 and 2025 were included involving 6,106 patients [23–62]. Common reasons for exclusion were unknown or selective LND based on pre- or intraoperative findings (*n* = 76) and the use of data from national databases with unknown or varying LND protocols (*n* = 25). Twenty-seven studies originated from Asia, twelve from Europe, and three from the United States of America.

This diagram demonstrates the systematic process of the search strategy and study selection. LND, lymph node dissection; PRISMA, preferred reporting items for systematic reviews and meta-analyses.

### Quality Assessment

The outcome of the quality assessment using risk-of-bias tools is presented in the Supplementary Figs. 1 to 4, with the overall risk of bias of each study reported in Table 1. The majority of the included studies demonstrated a moderate risk of bias (*n* = 22), with 18 studies regarded as having a serious risk of bias.

### Hepatocellular Carcinoma

#### Study Characteristics

Three studies described 677 patients who underwent resection of HCC between 2001 and 2010 (Table 1) [23–25]. Routine LND was performed in 601 patients (89%). One RCT by Wu et al. was included, in which 79 patients were grouped into hepatectomy alone or hepatectomy with LND and 41 patients (52%) underwent LND [23]. Mean tumor size ranged from 4.3 ± 2.4 to 7.0 ± 3.5 centimeters (Table 1) [23, 24]. Surgical and oncological outcomes are shown in Table 1. The specifications of LND (location / extend and number of LNs retrieved) are demonstrated in supplementary Table 1.

### Lymph Node Dissection and Outcome

**Fig. 2:**
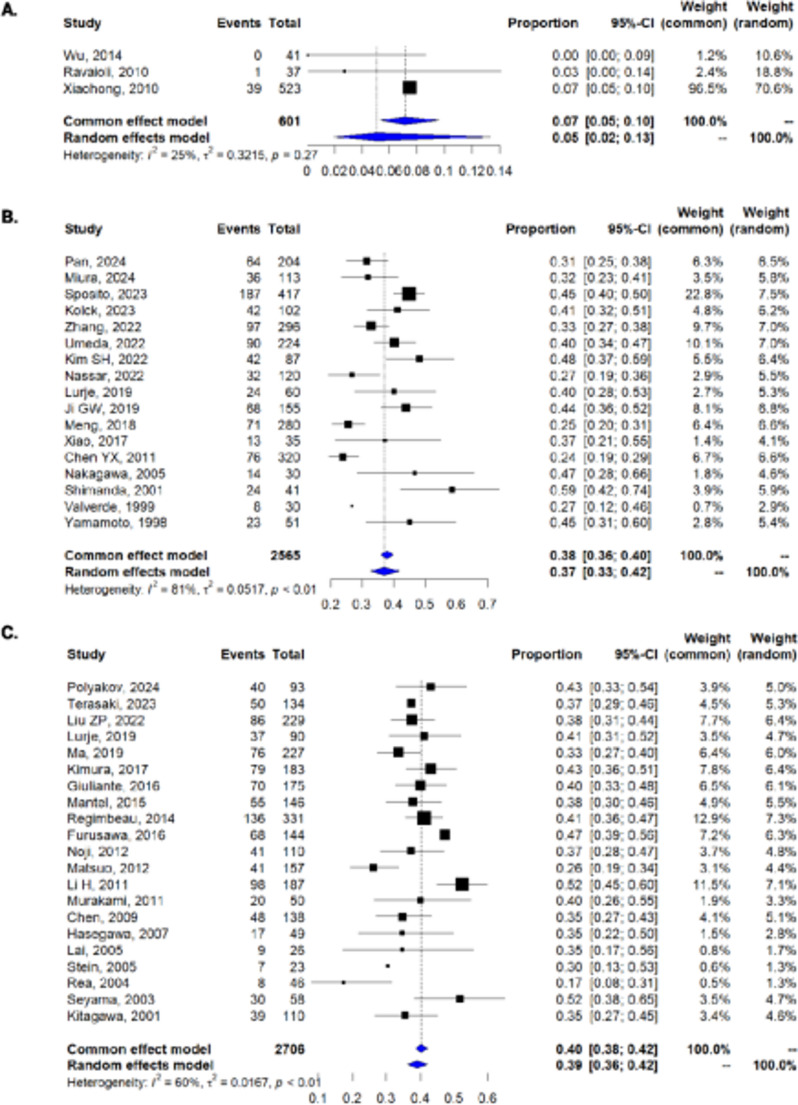
(**A**): Forest plot of the incidence of LNM in patients following routine LND during resection of HCC in three studies. (**B**): Forest plot of the incidence of LNM in patients following routine LND during resection of ICC in 17 studies. (**C**): Forest plot of the incidence of LNM in patients following routine LND during resection of PCC in 21 studies. CI, confidence interval; HCC, hepatocellular carcinoma; ICC, intrahepatic cholangiocarcinoma; LND, lymph node dissection; LNM, lymph node metastasis; PCC, perihilar cholangiocarcinoma

The incidence of LNM ranged from 0 to 8% [23–25]. with a pooled incidence of 5% (95% CI 2–13%, Fig. 2A). Mild heterogeneity was demonstrated (I^2^ = 25%, *p* = 0.27). Routine LND included retrieval of LNs from at least the hepatoduodenal ligament (supplementary Table 1) [23–25]. The mean number of retrieved LNs ranged from 2.8 ± 1.5 to 7.1 ± 2.7.

Two studies reported on differences in DFS and OS [23, 24] between LND + and LND- patients. The RCT by Wu et al. found no difference in 5-year DFS (63.4% vs. 63.2%, *p* = 0.811) [23]. and Ravaioli et al. also demonstrated no significant difference (34.0 vs. 33.0%, *p* > 0.05) [24]. Pooled survival analysis was not possible because fewer than three studies reported on DFS.

Wu et al. found comparable 5-year OS rates (70.7 vs. 65.8%, *p* = 0.881) [23], while the median OS and 5-year OS rate was significantly lower in LNM+ patients (28.0 months, 22% vs. 53.0 months, 43%, *p* = 0.003) in the study by Xiaohong et al. [25] Pooled survival analysis was not possible due to the lack of data.

### Intrahepatic Cholangiocarcinoma

#### Study Characteristics

Seventeen studies described 2,717 patients who underwent resection of ICC between 1980 and 2023 (Table 1) [26–42]. Routine LND was performed in 2,565 patients (94%). Reasons for omitting LND were preoperative misdiagnosis of HCC, liver dysfunction (not specified) or advanced disease diagnosed intraoperatively [37, 39, 42]. Mean tumor size ranged from 4.9 ± 2.1 to 10.5 ± 5.0 centimeters (Table 1B) [28, 30–33, 38, 41]. The R0 resection margin rate ranged from 50 to 100% [28, 31–34, 37, 42].

**Table 1:** Study characteristics and outcome of routine lymph node dissection

Author, year	Design	NA	ADJ	Tsize (cm)	R0 resection	Risk of bias	LND+	LND-	LNM	LNM Pooled	I^2^, %		OS Pooled, HR	I^2^, %
A. HCC										5% (2–13)	25%, *p* = 0.27		NA	**NA**
Wu 2014, [23]	RCT	NS	NS	LND+: 7.0 ± 3.5 LND-: 6.3 ± 2.9	79 (100)	Some	41 (52)	38 (48)	0 (0)					
Ravaioli 2010, [24]	P	NS	NS	LND+: 4.7 ± 3.2 LND-: 4.3 ± 2.4	NS	Serious	37 (49)	38 (51)	1 (3					
Xiaohong 2010, [25]	R	NS	NS	NS	NS	Moderate	523 (100)	0 (0)	39 (8)					
B. ICC										37% (33–42)	81%, *p* < 0.01		2.46 (1.66–3.65)	90%,*p* < 0.01
Pan 2024, [26]	R	0 (0)	NS	NS	NS	Serious	204 (100)	0 (0)	64 (31)					
Miura 2024, [27]	R	0 (0)	NS	NS	NS	Moderate	113 (100)	0 (0)	36 (32)					
Sposito 2023, [28]	R	NS	302 (72)	5.4 (4.0,7.4)	350 (84)	Serious	417 (100)	0 (0)	187 (45)					
Kolck 2023, [29]	R	NS	NS	NS	NS	Moderate	102 (100)	0 (0)	42 (42)					
Zhang 2022, [30]	R	0 (0)	NS	5.0 (0.5–14.0)	NS	Moderate	296 (100)	0 (0)	97 (33)					
Umeda 2022, [31]	R	8 (3)	125 (40)	LND+: 4.5 (3.9) LND-: 3.3 (3.2)	310 (100)	Serious	224 (72)	86 (28)	90 (40)					
Kim SH 2022, [32]	R	7 (8)	43 (49)	4.9 ± 2.1	87 (100)	Serious	87 (100)	0 (0)	42 (48)					
Nassar 2022, [33]	R	NS	41 (34)	6.6 ± 3.8	97 (81)	Serious	120 (100)	0 (0)	32 (27)					
Lurje 2019,[34]	R	0 (0)	34 (48)	NS	62 (87)	Serious	60 (85)	11 (15)	24 (40)					
Ji GW 2019, [35]	R	NS	NS	NS	NS	Moderate	155 (100)	0 (0)	68 (44)					
Meng 2018, [36]	R	0 (0)	NS	NS	NS	Moderate	280 (100)	0 (0)	71 (26)					
Xiao 2017, [37]	R	NS	NS	NS	42 (86)	Serious	35 (71)	14 (29)	13 (37)					
Chen YX 2011, [38]	R	NS	NS	6.1 ± 2.9	NS	Moderate	320 (100)	0 (0)	76 (24)					
Nakagawa 2005, [39]	R	NS	NS	NS	NS	Serious	30 (68)	14 (32)	14 (47)					
Shimada 2001, [40]	R	NS	NS	NS	NS	Moderate	41 (84)	8 (16)	24 (59)					
Valverde 1999, [41]	R	NS	15 (50)	10.5 ± 5.0	NS	Serious	30 (100)	0 (0)	8 (27)					
Yamamoto 1998, [42]	R	0 (0)	0 (0)	NS	35 (50)	Moderate	51 (73)	19 (27)	23 (45)					
C. PCC										39% (36–42)	60%, *p* < 0.01		2.01 (1.74–2.33	45%,*p* = 0.02
Polyakov 2024, [43]	R	NS	47 (48)	NS	66 (67)	Moderate	93 (95)	5 (5)	40 (43)					
Terasaki 2023, [44]	R	NS	0 (0)	NS	121 (90)	Serious	134 (100)	0 (0)	50 (37)					
Liu ZP 2022, [45]	R	NS	0 (0)	3.0 (2.0-3.8)	229 (100)	Moderate	229 (100)	0 (0)	86 (38)					
Lurje 2019, [34]	R	0 (0)	37 (41)	NS	76 (84)	Serious	90 (99)	1 (1)	37 (41)					
Ma 2019, [46]	R	NS	27 (12)	NS	179 (79)	Serious	227 (100)	0 (0)	76 (34)					
Kimura 2017, [47]	R	NS	67 (37)	NS	112 (61)	Moderate	183 (100)	0 (0)	79 (43)					
Giuliante 2016, [48]	R	NS	75 (43)	2.7 ± 1.2	143 (82)	Moderate	175 (100)	0 (0)	70 (40)					
Mantel 2015, [49]	R	NS	NS	NS	NS	NS	146 (100)	0 (0)	55 (38)					
Regimbeau 2014, [50]	R	0 (0)	73 (22)	2.3 (1.0–4.0)	195 (59)	Serious	331 (100)	0 (0)	136 (41)					
Furusawa 2014, [51]	R	0 (0)	22 (15)	NS	107 (74)	Moderate	144 (100)	0 (0)	68 (47)					
Noji 2012, [52]	R	NS	0 (0)	NS	94 (85)	Moderate	110 (100)	0 (0)	41 (37)					
Matsuo 2012, [53]	R	NS	NS	3.0 ± 2.2	120 (76)	Moderate	157 (100)	0 (0)	41 (26)					
Li H 2011, [54]	R	NS	NS	NS	141 (75)	Moderate	187 (100)	0 (0)	98 (52)					
Murakami 2011, [55]	R	NS	27 (54)	NS	37 (74)	Serious	50 (100)	0 (0)	20 (40)					
Chen XP 2009, [56]	P	NS	0 (0)	NS	123 (89)	Moderate	138 (100)	0 (0)	48 (35)					
Hasegawa 2007, [57]	R	NS	0 (0)	NS	38 (78)	Moderate	49 (100)	0 (0)	17 (35)					
Lai 2005, [58]	R	NS	0 (0)	3.4 (1.1-5.0)	19 (73)	Serious	26 (100)	0 (0)	9 (35)					
Stein 2005, [59]	R	NS	NS	NS	NS	Serious	23 (100)	0 (0)	7 (30)					
Rea 2004, [60]	R	NS	19 (41)	2.9 ± 1.8	37 (80)	Moderate	46 (100)	0 (0)	8 (17)					
Seyama 2003, [61]	R	NS	NS	NS	37 (64)	Moderate	58 (100)	0 (0)	30 (52)					
Kitagawa 2001, [62]	R	NS	NS	NS	110 (100)	Serious	110 (100)	0 (0)	39 (36)					

Values are n (%) unless otherwise indicated. * Data are mean ± standard deviation, median (range) or median (interquartile range)

*ADJ* adjuvant treatment, *cm*, centimeters, *I*^2^ heterogenity, *HCC* hepatocellular carcinoma, *ICC* intrahepatic cholangiocarcinoma, *LND* lymph node dissection, *LND+* lymph node dissection, *LND-* no lymph node dissection, *LNM pooled* pooled incidence of LNM (%) with 95% confidence interval, *NA* neoadjuvant treatment, *NS* not specified, *OS pooled* pooled overall survival (hazard ratio) with 95% confidence interval, *P* prospective study design, *PCC* perihilar cholangiocarcinoma, *R* retrospective study design, *R0* resection with microscopically negative margins, *RCT* randomized controlled trial, *Tsize* tumor size

### Lymph Node Dissection and Outcome

The incidence of LNM ranged from 24 to 59% (Table 1) [26–42], with a pooled incidence of 37% (95% CI 33–42%, Fig. 2B). Substantial heterogeneity was found (I^2^ = 81%, *p* < 0.01). Meta-regression did not identify significant study factors explaining between study-heterogeneity (supplementary Table 2). The funnel plot was symmetric and Egger’s test was non-significant (supplementary Fig. 5). Leave-one-out sensitivity analysis confirmed the robustness of the pooled estimate (range 35.9–38.2).

Routine LND included retrieval of LNs from at least the hepatoduodenal ligament [26–42]. Five studies used distinct LND protocols based on left or right tumor location [27, 28, 37, 39, 42], with the hepatoduodenal ligament being the most common sight of LND (supplementary Table 1) [31, 38, 39, 42]. The median number of retrieved LNs ranged from 6 to 12 [27, 28, 32, 37]. 

Six studies reported on DFS [28, 30, 32–35], ranging from 16.0 to 24.3 months [28, 30, 33, 35] Sposito et al. demonstrated superior median DFS in LNM+ patients who received adequate LND with ≥ 6 retrieved LNs compared to patients with inadequate or no LND (13 months vs. 9 months, *p* = 0.008) [28]. For LNM- patients, this difference was 43 versus 22 months (*p* = 0.029) [28]. Three studies found significantly worse median DFS in LNM+ patients [28, 32, 34]. and in three studies, LNM was an independent risk factor for worse DFS [30, 35, 41]. In LNM+ patients, the number of LNM was an independent predictor for worse DFS [32]. Pooled survival analysis could not be performed.

**Fig. 3:**
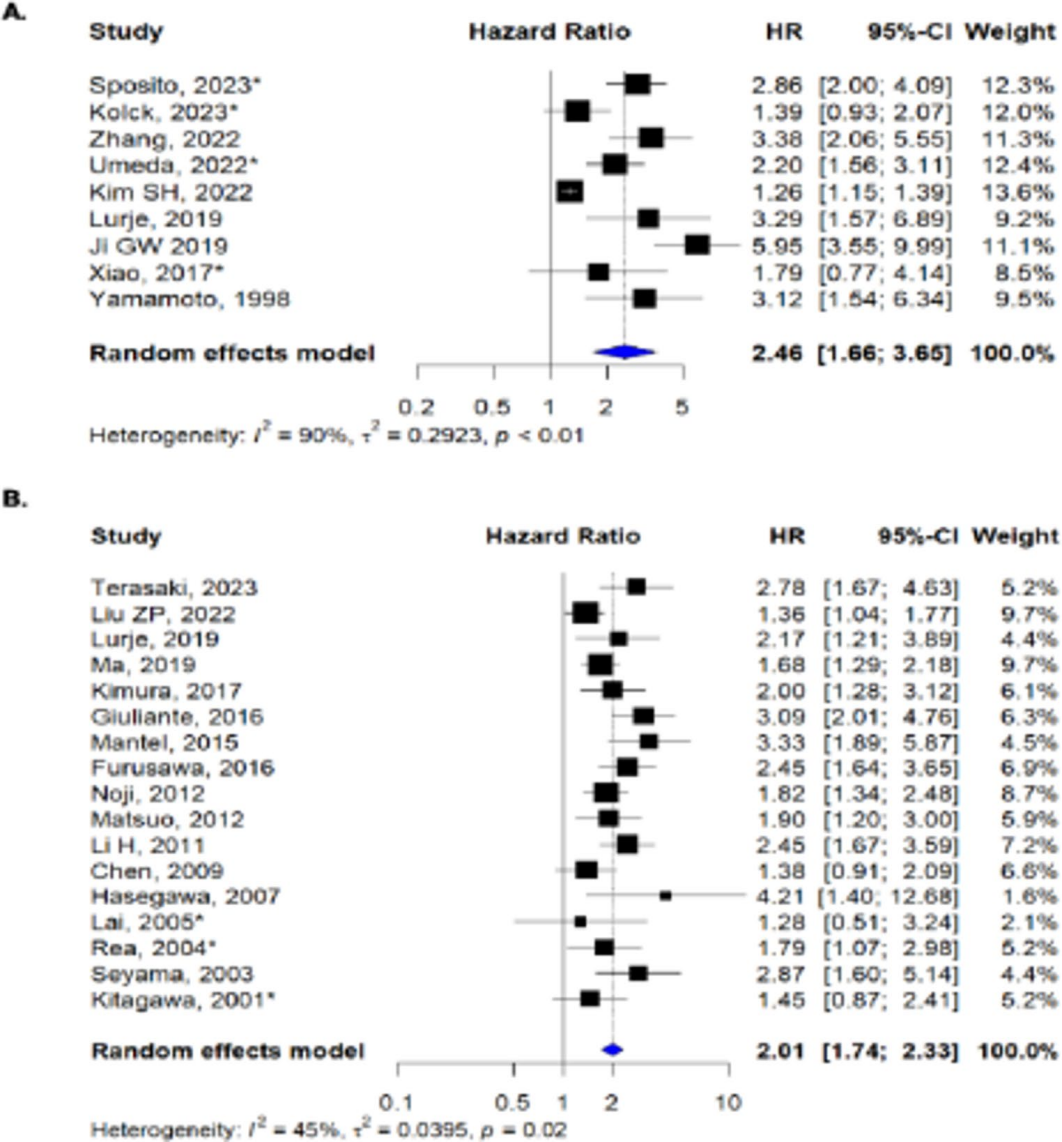
(**A**): Forest plot of the hazard ratios comparing OS between patients with and without LNM following routine LND during ICC resection in nine studies. (**B**): Forest plot of the HRs comparing OS between patients with and without LNM following routine LND during PCC resection in 17 studies. The asterisk (*) symbol represents studies in which the HR and 95% CI were estimated based on extracted data with WebPlotDigitizer 4.6. CI, confidence interval; HR, hazard ratio; ICC, intrahepatic cholangiocarcinoma; LND, lymph node dissection; LNM, lymph node metastasis; PCC, perihilar cholangiocarcinoma

Ten studies reported on OS [28, 30–35, 37, 41, 42], ranging from 28.0 to 55.0 months following routine LND during resection [28, 30, 31, 33, 35, 37, 41]. Sposito et al. reported superior median OS in LNM+ patients who underwent adequate LND with ≥ 6 retrieved LNs compared to patients with inadequate or no LND (28 months vs. 23 months, *p* = 0.023) [28]. For LNM- patients, this difference was 68 versus 44 months (*p* = 0.003) [28]. Two retrospective studies confirmed superior median OS and 5-year OS rates in LND+ patients [31, 37], with one study demonstrating LND to be independently associated with improved OS [31]. Five studies found significantly worse median OS in 358 LNM+ patients compared to 461 LNM- patients [28, 31, 32, 34, 37]. The retrospective analysis by Kim SH et al. found worse median OS with an increasing number of LNMs [32]. Pooled analysis of OS in LNM- and LNM+ patients demonstrated that the presence of LNM was associated with worse OS (HR 2.46, 95% CI 1.66–3.65, Fig. 3A) [28–32, 34, 35, 37, 42]. Substantial heterogeneity was found (I^2^ = 90%, *p* < 0.01). Meta-regression identified no significant study factors (supplementary Table 2). Egger’s test was not performed because the test is unreliable with fewer than 10 studies. Leave-one-out sensitivity analysis demonstrated that the pooled HR was robust across all iterations (range 2.18–2.72).

### Perihilar Cholangiocarcinoma

#### Study Characteristics

Twenty-one studies described 2,712 patients who underwent resection of PCC between 1979 and 2023 (Table 1C) [34, 43–62]. Routine LND was performed in 2,706 patients (99.8%) 34,43 Median tumor size ranged from 2.3 (1.0–4.0) to 3.4 (1.1-5.0) centimeters (Table 1) [45, 48, 50, 53, 58, 60]. and the R0 resection margin rate ranged from 61 to 100% [34, 43–48, 50–58, 60–62]. 

### Lymph Node Dissection and Outcome

The incidence of LNM ranged from 17 to 52% (Table 1) [34, 43–62]., with a pooled incidence of 39% (95% CI 36–42%, Fig. 2C). Substantial heterogeneity was found (I^2^ = 60%, *p* < 0.01). Meta-regression did not identify any significant study factors (supplementary Table 2). Egger’s test was statistically significant (*p* = 0.015) but the negative estimate (-2.30) indicates an opposite direction of what classical publication bias would predict and is more consistent with case-mix heterogeneity (supplementary Fig. 5). Leave-one-out sensitivity analysis confirmed the robustness of the pooled estimate (range 38.4–39.9%).

Routine LND included retrieval of LNs from at least the hepatoduodenal ligament, with a median number of retrieved LNs ranging from 4 to 17 (supplementary Table 1) [43–50, 55]. The most common region of LNM was the hepatoduodenal ligament [44, 46, 53, 62]. 

Five studies reported on DFS [34, 43, 45, 46, 50], ranging from 16.0 to 36.0 months [34, 45, 46]. Two studies found a significantly worse median DFS in LNM+ patients [34, 45], with a significantly lower 5-year DFS rate in LNM+ patients (10.7 vs. 20.6%, *p* = 0.001) [45]. Liu ZP et al. identified LNM as an independent prognostic factor for worse DFS [45]. Pooled survival analysis was not possible. Twenty studies reported on OS [34, 43–58, 60–62], ranging from 20.0 to 45.5 months [34, 45–47, 53, 55, 57, 58, 60]. Five-year OS ranged from 12.0 to 53.0% in LND+ patients [45–48, 50, 52–55, 57, 58, 60, 61] and retrieving 1–5 LNs during routine LND resulted in a lower 5-year OS rate (34.2%) compared to 6–7 LNs (64.5%) or ≥ 8 LNs (62.7%, *p* = 0.047) [48]. In eight studies, the 5-year OS rate was significantly lower in LNM+ patients [44, 45, 47, 48, 51, 54, 60, 61]. Seyama et al. and Kitagawa et al. demonstrated thatLNM beyond the hepatoduodenal ligament was associated with worse OS [61, 62]. Based on 17studies describing 2,109 patients, pooled analysis demonstrated that LNMs was associated with worse OS (HR 2.01, 95% CI 1.74–2.33, Fig. 3B) 34,44–49,51–54,56–58,60–62] were eligible for pooled analysis of OS in LNM- and LNM+ patients. Moderate heterogeneity was found (I^2^ = 45%, *p* = 0.02). Meta-regression yielded no significant study factors (supplementary Fig. 5). Egger’s test was borderline (*p* = 0.050) (supplementary Fig. 5). Leave-one-out sensitivity analysis confirmed the robustness of the pooled HRs (range 1.94–2.08).

## Discussion

This systematic review and meta-analysis pooled data from 40 studies involving 5,872 patients who underwent routine LND independently of pre- or intraoperative suspicion of LNM during resection of HCC, ICC and PCC. The pooled incidence of LNM following routine LND was 5% among 601 HCC patients, 37% among 2,565 ICC patients, and 39% among 2,706 PCC patients. Pooled survival analysis was available for ICC and PCC patients and found that the presence of LNM was associated with worse OS.

Since the proportion of patients eligible for surgical resection is low, data on outcomes following LND during resection are rare and usually derive from retrospective, small single-center studies [4, 9, 10, 12–15]. Previous systematic reviews and meta-analyses were limited by studies that performed selective LND based on pre- or intraoperative suspicion of LNM during resection [4, 9, 10, 12–15]. However, conventional preoperative imaging modalities are not accurate in detecting LNM and selecting patients for LND. Consistent with this, two of the included studies found that 39% [26] and 51% [35] of patients without suspicion of LNM on preoperative CT or MRI imaging had LNM on histological examination following routine LND during ICC resection. To our knowledge, we present the first meta-analysis that analyzes the incidence of LNM and survival outcomes in a large cohort of patients undergoing routine LND independently of pre- or intraoperative findings during resection of HCC, ICC, and PCC.

A pooled LNM incidence of 5% was found among patients who underwent resection for HCC, which agrees with a population-based cancer registry study describing an incidence of 1–5% [63]. However, the incidence reported by our group is significantly lower than the incidence of 45% reported in a previous meta-analysis by Amini et al. [4] although the latter included studies with small sample sizes and studies performing selective rather than routine LND. [4] The heterogeneity of the reported incidence of LNM may also be explained by different resected LN stations. Regarding the association between LND and survival, the 5-year DFS and OS rates were not significantly different in LND + and LND- patients [23, 24]. Few available studies, small sample sizes, and absence of data on (neo)adjuvant therapy introduce risks of bias in the present analysis. Overall, our data support the current guidelines which recommend not to perform routine LND during HCC resection [2, 8]. 

With regards to routine LND in resection of ICC, we demonstrated a pooled incidence of LNM of 37% based on 17 included studies This is in line with three previous systematic reviews reporting an incidence of 28–45% [4, 13, 14]. Median OS and 5-year OS rate following routine LND ranged from 28 to 55 months and 20–43%, respectively. Despite R0 resections in most patients (median 85%), these data confirm the relatively poor prognosis likely caused by the aggressive tumor biology and late diagnosis with resection of large tumors (mean tumor size: 4.9–10.5 cm). Previous meta-analyses found no survival benefits of LND [9, 12, 13]. However, their results must be interpreted with caution because studies performing selective rather than routine LND were included. In fact, recent studies suggested superior long-term survival in clinically node-negative ICC patients following routine LND compared to no or inadequate (< 6 LNs) LND [16–18]. Our systematic review also found trends of improved OS in patients following routine LND [28, 31, 37]. With a high incidence in ICC, the presence of LNM was associated with worse OS in pooled survival analysis. These data support the current guidelines to perform routine LND during resection of ICC.

Based upon 21 studies describing a total of 2,706 patients who underwent routine LND during PCC resection, meta-analysis demonstrated a pooled incidence of LNM of 39%. This finding is comparable to the reported incidence of 31–58% in a systematic review by Kambakamba et al. [15] Median OS and 5-year OS rate ranged from 20 to 45 months and 12–53%, respectively. Despite the fact that most of the included patients (median 78%) underwent R0 resections, the prognosis remained poor, again emphasizing the aggressive tumor biology of PCC. A previous systematic review found no survival benefits of LND, but it involved only one study with 22 patients undergoing selective LND [10]. Our group found trends of improved survival outcomes in patients following routine LND.[48.49] With a high incidence in PCC, the presence of LNM was associated with worse OS in pooled survival analysis. The discrepancy between the relatively high percentage of R0 resections but relatively poor overall survival is, at least partly, explained by the high incidence of LNM’s, which has been supported by recent studies demonstrating that the presence of LNMs has a far greater influence on OS compared to radicality [64]. 

The ideal extent of LND remains unknown. The latest TNM classification recommends the removal of ≥ 15 LNs during resection of PCC [65]. However, three out of ten PCC studies reporting the number of retrieved LNs met this recommendation [44, 55, 62]. Two previous systematic reviews also found that few studies dissected ≥ 15 LNs, which is in line with our own practice in treating these patients [10, 15]. Regarding the effect of retrieved LN count on prognosis, Giuliante et al. demonstrated that dissection of 6–7 LNs and ≥ 8 LNs compared to 1–5 LNs significantly improved the 5-year OS rate in PCC patients [48]. Polyakov et al. reported that retrieving ≥ 6 LNs was associated with superior survival [43]. However, some physicians advocate that extending the amount of LN’s being dissected, might be associated with a higher morbidity routine, due to chyle leakage. Regarding the risks of routine LND, a recent meta-analysis by Yeow et al. found no different rates of major complications in 1020 LND+ patients compared to 753 LND- patients following ICC resection [9]. 

Several limitations of the present meta-analysis should be acknowledged. First, most included studies had a retrospective, small single-center study design, which introduces risks of confounding and selection bias. The risk of selection bias was reduced by including only studies that performed routine LND independently of pre- and intraoperative suspicion of LNM. However, substantial heterogeneity was present in several pooled analysis. Meta-regression did not identify study-level sources of heterogeneity. The residual heterogeneity may therefore reflect differences in other factors not captured at the study level. Many studies had inclusion periods that extended over ten years, with the risk of changes in treatment protocols and expertise. Also, different approaches to LND and histological LN examination among studies may impact the generalizability of the outcomes. Finally, neoadjuvant or adjuvant therapy was not specified in most studies or differed among studies, which introduces potential heterogeneity. Another limitation includes the use of univariable HRs across the survival analyses. As multivariable HRs were not always available and a proportion of the hazard ratios had to be reconstructed from published Kaplan–Meier curves, univariable HRs were used for consistency. As a result, the pooled HRs should be interpreted as unadjusted prognostic associations rather than independent prognostic effects, and residual confounding cannot be excluded. Sensitivity analyses did not show systematic differences between reconstructed and directly reported HRs.

## Conclusion

Routine LND during resection of HCC is associated with a low incidence of LNM and does not improve survival. The high incidence of LNM and trends of improved survival following routine LND during resection of ICC and PCC justify the current recommendation to perform routine LND in the treatment of ICC and PCC. Following routine LND during resection of ICC and PCC, the presence of LNM is associated with a two-fold increased risk of death. Future research should target the identification of novel strategies that lead to superior preoperative detection of LNM. New strategies being investigated include endoscopic ultrasound with tissue acquisition (EUS-TA) of lymph nodes in patients with potentially resectable ICC, de Jong et al. [66] and the fibroblast-activating protein (FAP) inhibitors as novel PET/CT-tracers (FAPI-PET/CT) in the staging of patients with cholangiocarcinoma and HCC [67]. Furthermore, we should focus on identifying segmental liver lymphatic drainage patterns to determine the ideal extent of LND based on tumor location and which patients with LNM following routine LND will benefit most from adjuvant or targeted systematic therapy.

## Supplementary Information

Below is the link to the electronic supplementary material.

Supplementary figure 1. Risk of bias assessment of randomised controlled trial in hepatocellular carcinoma

Supplementary figure 2. Risk of bias assessment of nonrandomised studies in hepatocellular carcinoma

Supplementary figure 3. Risk of bias assessment of nonrandomised studies in intrahepatic cholangiocarcinoma

Supplementary figure 4. Risk of bias assessment of nonrandomised studies in perihilar cholangiocarcinoma

Supplementary figure 5. Publication bias assessment in intrahepatic and perihilar cholangiocarcinoma

Supplementary Table 1

Supplementary Table 2

## Data Availability

No datasets were generated or analysed during the current study.
