# Supplementary material for: Routine Lymph Node Dissection in the Surgical Treatment of Primary Liver Tumors: a Systematic Review and Meta-Analysis
Source: J Gastrointest Cancer. 2026 Jul 16;57(1):154. doi: 10.1007/s12029-026-01516-9 (PMC13375768; doi:10.1007/s12029-026-01516-9)
Supplement: Supplementary file 1 — Supplementary figure 1. Risk of bias assessment of randomised controlled trial in hepatocellular carcinoma [file 12029_2026_1516_MOESM1_ESM.docx]

**Supplementary figure 1.** Risk of bias assessment of randomised controlled trial in hepatocellular carcinoma

**
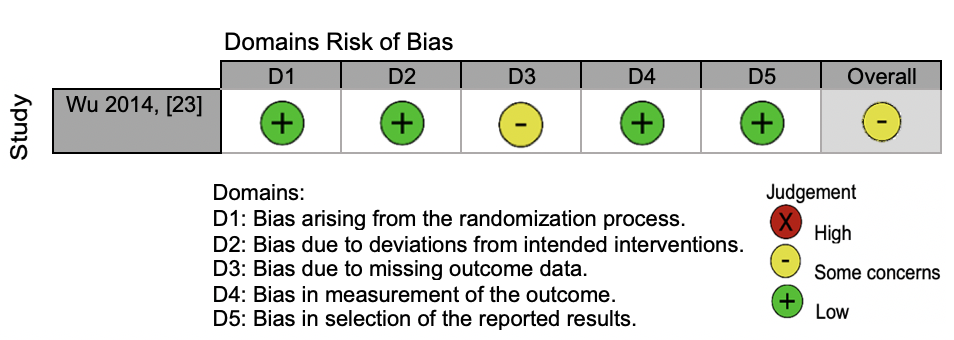
**

The risk of bias in one randomized controlled trial on HCC patients was assessed with the risk-of-bias (RoB-2) tool across five domains.

*HCC* hepatocellular carcinoma.
