# Supplementary material for: Routine Lymph Node Dissection in the Surgical Treatment of Primary Liver Tumors: a Systematic Review and Meta-Analysis
Source: J Gastrointest Cancer. 2026 Jul 16;57(1):154. doi: 10.1007/s12029-026-01516-9 (PMC13375768; doi:10.1007/s12029-026-01516-9)
Supplement: Supplementary file 2 — Supplementary figure 2. Risk of bias assessment of nonrandomised studies in hepatocellular carcinoma [file 12029_2026_1516_MOESM2_ESM.docx]

**Supplementary figure 2.** Risk of bias assessment of nonrandomised studies in hepatocellular carcinoma

**B.
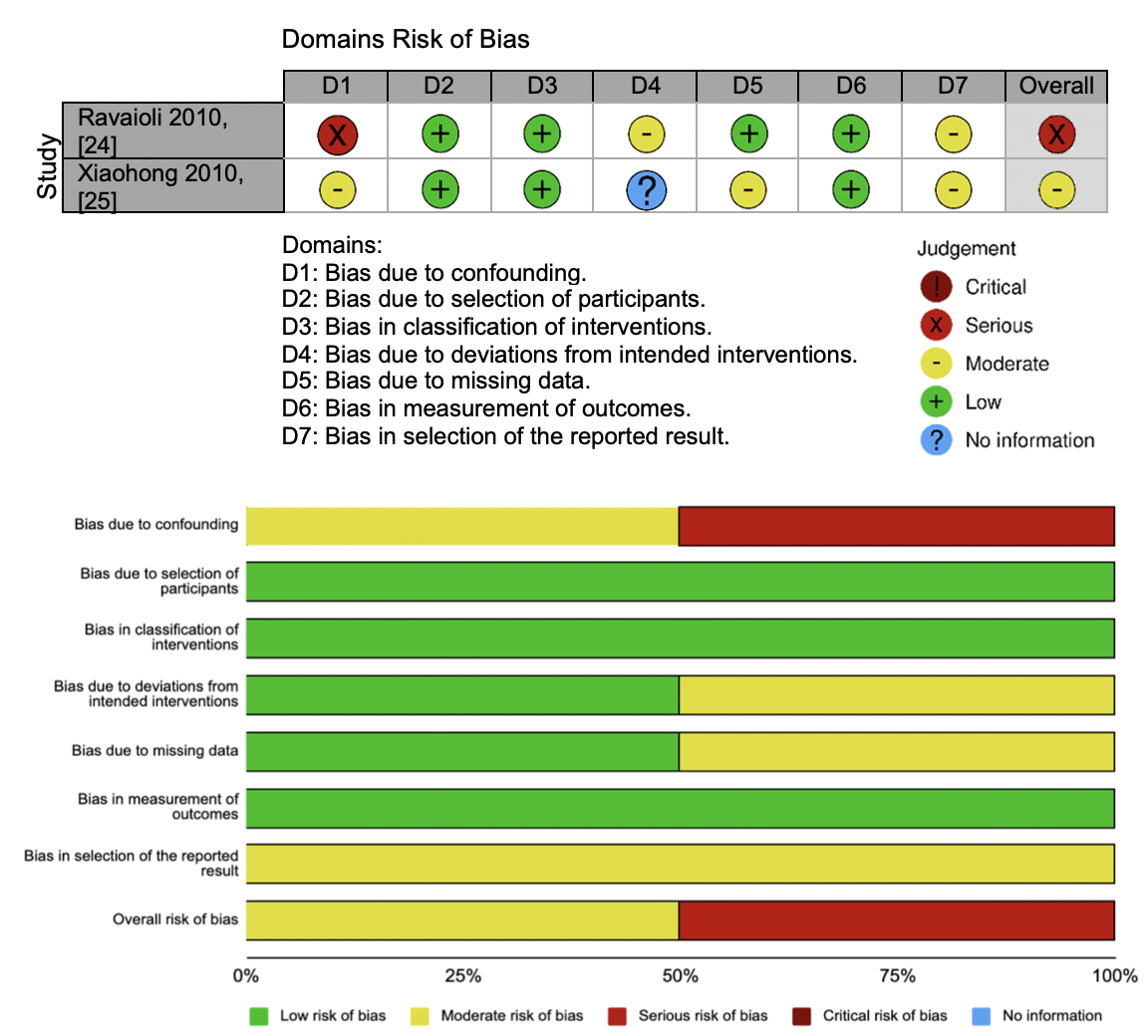
**

**A.**

(A): The risk of bias in two nonrandomised studies on HCC patients was assessed with the ROBINS-I tool across seven domains. (B): Summary of the risk of bias in each domain of the ROBINS-I tool.

*HCC* hepatocellular carcinoma, *ROBINS-I* risk of bias in nonrandomised studies-interventions.
