# Supplementary material for: Routine Lymph Node Dissection in the Surgical Treatment of Primary Liver Tumors: a Systematic Review and Meta-Analysis
Source: J Gastrointest Cancer. 2026 Jul 16;57(1):154. doi: 10.1007/s12029-026-01516-9 (PMC13375768; doi:10.1007/s12029-026-01516-9)
Supplement: Supplementary file 4 — Supplementary figure 4. Risk of bias assessment of nonrandomised studies in perihilar cholangiocarcinoma [file 12029_2026_1516_MOESM4_ESM.docx]

**Supplementary figure 4.** Risk of bias assessment of nonrandomised studies in perihilar cholangiocarcinoma

**A.**
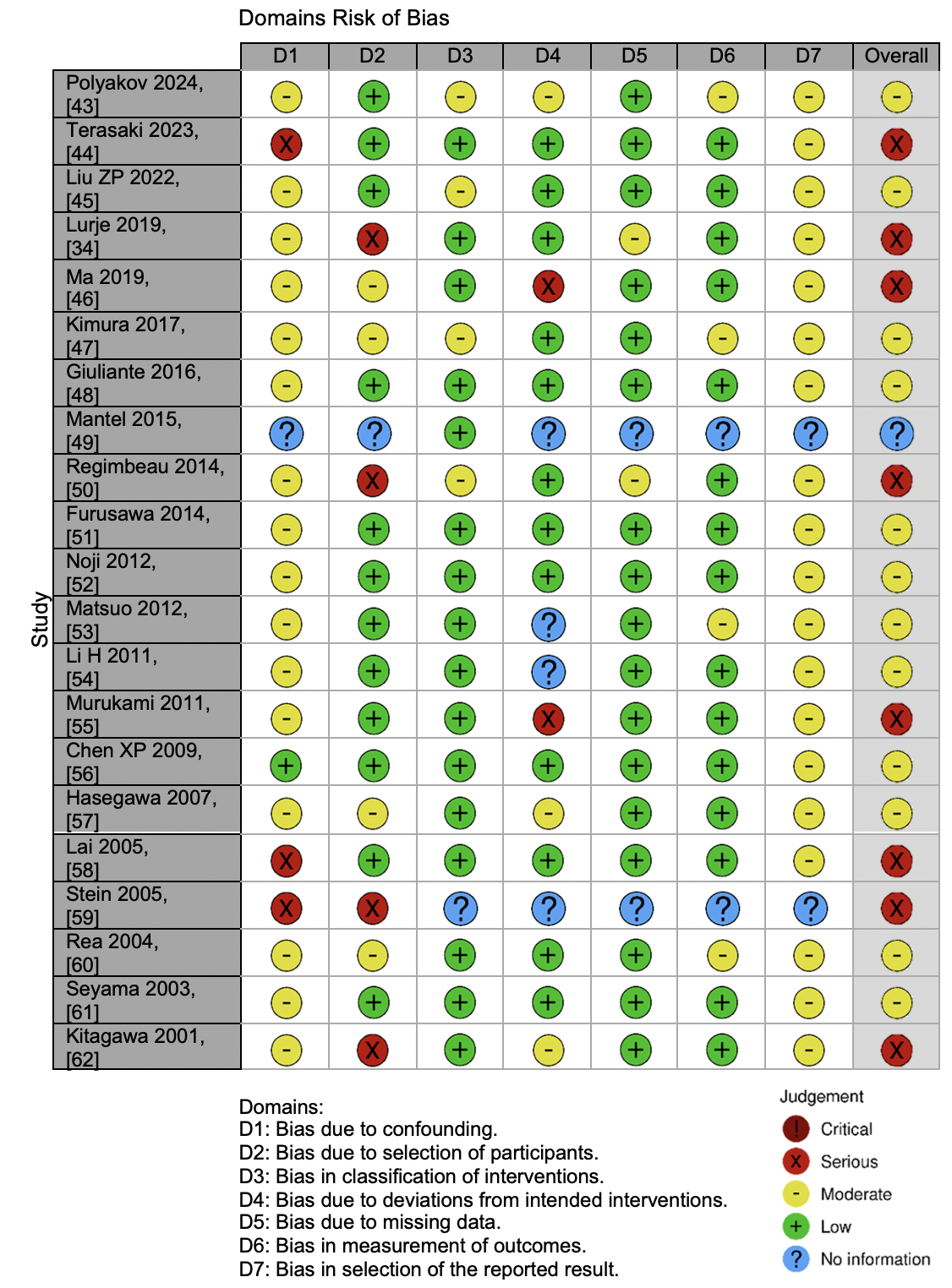


**B.**
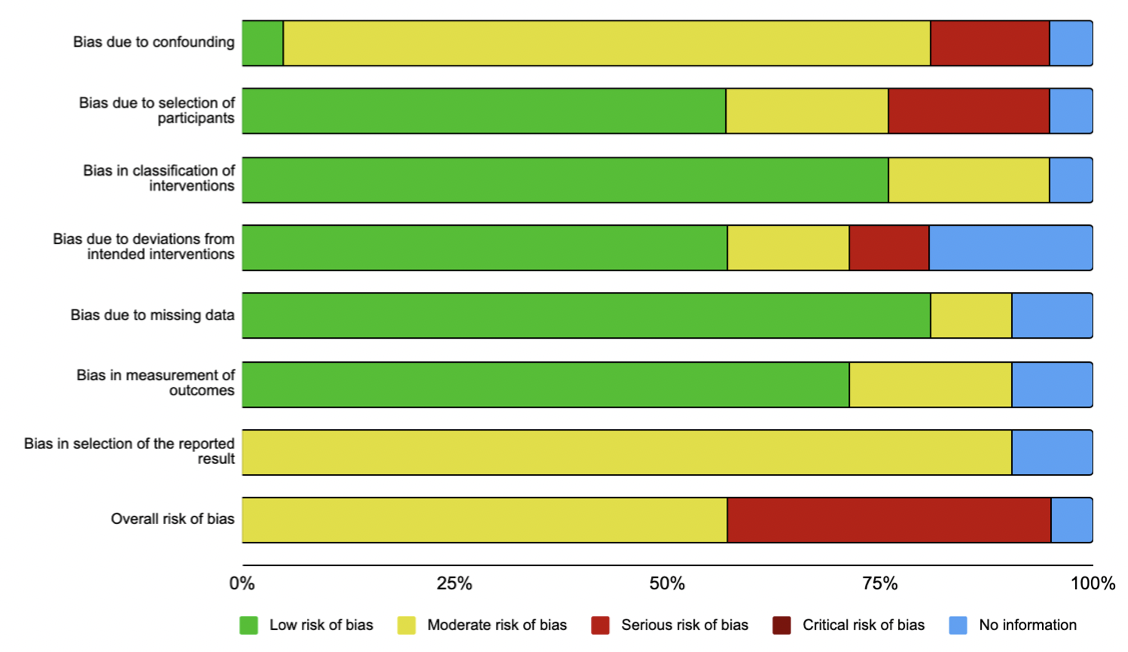


(A): The risk of bias in 21 nonrandomised studies on PCC patients was assessed with the ROBINS-I tool across seven domains. (B): Summary of the risk of bias in each domain of the ROBINS-I tool.

*PCC* perihilar cholangiocarcinoma, *ROBINS-I* risk of bias in nonrandomised studies-interventio
