# Supplementary material for: Routine Lymph Node Dissection in the Surgical Treatment of Primary Liver Tumors: a Systematic Review and Meta-Analysis
Source: J Gastrointest Cancer. 2026 Jul 16;57(1):154. doi: 10.1007/s12029-026-01516-9 (PMC13375768; doi:10.1007/s12029-026-01516-9)

**Supplementary figure 5.** Publication bias assessment in intrahepatic and perihilar cholangiocarcinoma


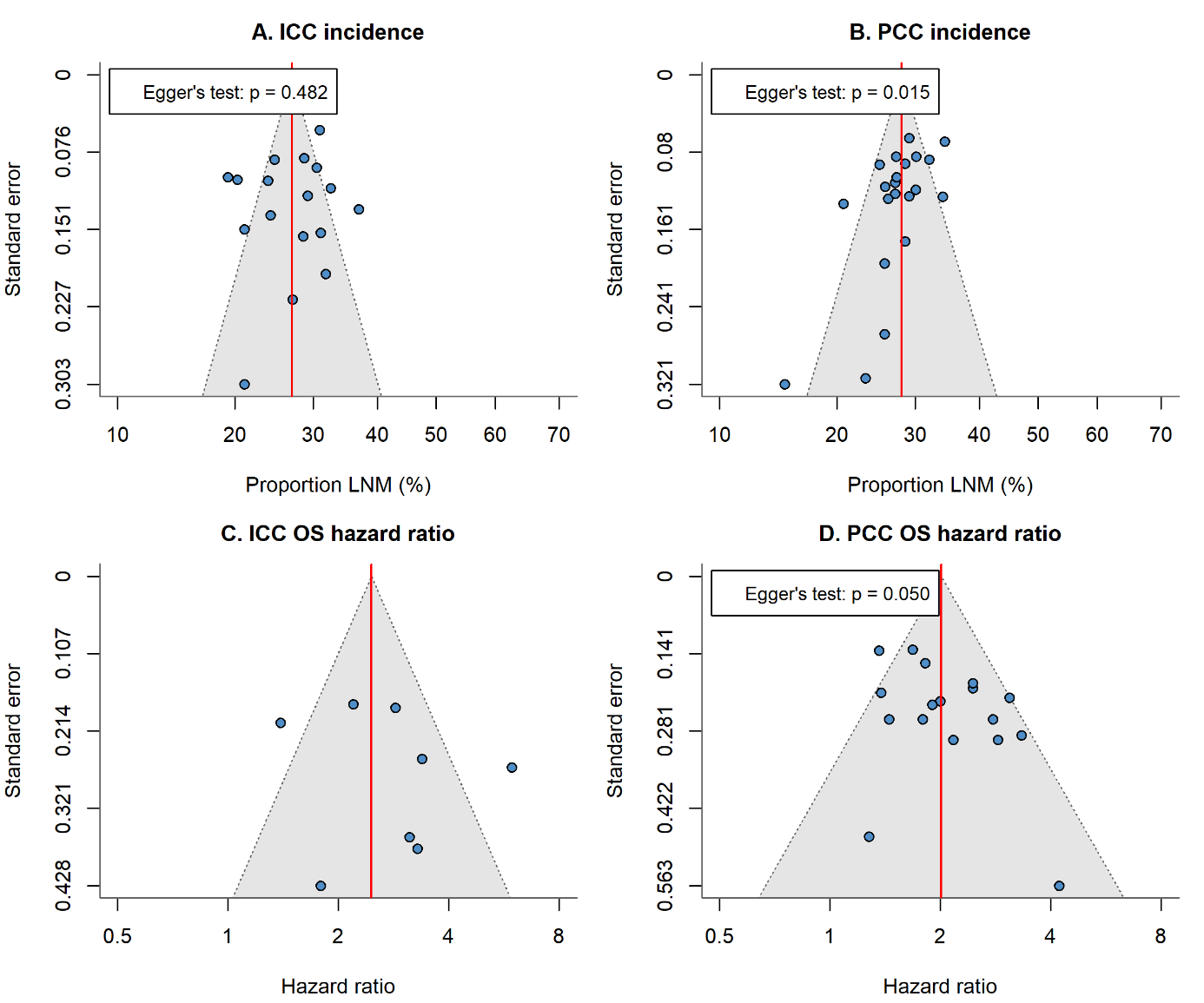

Supplement: Supplementary file 5 — Supplementary figure 5. Publication bias assessment in intrahepatic and perihilar cholangiocarcinoma [file 12029_2026_1516_MOESM5_ESM.docx]
