# Supplementary material for: Routine Lymph Node Dissection in the Surgical Treatment of Primary Liver Tumors: a Systematic Review and Meta-Analysis
Source: J Gastrointest Cancer. 2026 Jul 16;57(1):154. doi: 10.1007/s12029-026-01516-9 (PMC13375768; doi:10.1007/s12029-026-01516-9)
Supplement: Supplementary file 6 — Supplementary Table 1 [file 12029_2026_1516_MOESM6_ESM.docx]

| Author, publication year | Definition LND | LND location | Frequent LNM region | *LN total |  |
| --- | --- | --- | --- | --- | --- |
| **A. HCC** |  |  |  |  |  |
| Wu 2014,^23^ | Preventive regional LND | HDL, CHA | NA | 2.8 ± 1.5 |  |
| Ravaioli 2010,^24^ | Regional LND with ≥4 LNs without radiological LNM evidence | HDL, CHA | NS | 6.7 ± 4.8 |  |
| Xiaohong 2010,^25^ | Routine regional LND | HDL, PPH | HDL | 7.1 ± 2.7 |  |
| **B. ICC** |  |  |  |  |  |
| Pan 2024,^26^ | Curative-intent resection with LND | NS | NS | NS |  |
| Miura 2024,^27^ | Systematic LND with ≥6 LNs | HDL, peri-pancreatic LNs.  Left: plus gastro-hepatic LNs | NS | 12 (6-39) |  |
| Sposito 2023,^28^ | Routine LND with ≥6 LNs | HDL, CHA. Left: plus station 7. Right: plus station 13 | NS | 10 (7, 13) |  |
| Kolck 2023,^29^ | Hilar LND with ≥6 LNs | NS | NS | NS |  |
| Zhang 2022,^30^ | Regional LND | HDL, PPH, para-aortic LNs | NS | NS |  |
| Umeda 2022,^31^ | Regional systematic LND | Peri-hilum to HDL | HDL | NS |  |
| Kim SH 2022,^32^ | LND with ≥5 harvested LNs | HDL, CHA | NS | 11 (9-18) |  |
| Nassar 2022,^33^ | Curative-intent surgery with LND | HDL, celiac axis | NS | NS |  |
| Lurje 2019,^34^ | Routine LND | HDL, CHA, PPH, celiac axis | NS | NS |  |
| Ji GW 2019,^35^ | Curative-intent resection with LND | NS | NS | NS |  |
| Meng 2018,^36^ | Resection with regional LND | NS | NS | NS |  |
| Xiao 2017,^37^ | Resection with regional LND | HDL, CHA, celiac axis. Left: plus station 1,3,7. Right: plus station 13 | NS | 6 (3-15) |  |
| Chen YX 2011,^38^ | LND in all patients | HDL, CHA, PPH, para-aortic, greater omental LNs | HDL | NS |  |
| Nakagawa 2005,^39^ | Portal LND | HDL, CHA, PPH, celiac axis. Left: plus station 1,3,7 | HDL | NS |  |
| Shimada 2001,^40^ | Hepatic resection with LND | HDL, CHA | NS | NS |  |
| Valverde 1999,^41^ | Routine LND | Portal triad | NS | NS |  |
| Yamamoto 1998,^42^ | Routine LND | HDL, CHA, PPH, celiac axis, LGA. Left: plus right cardiac region, lesser curvature stomach. | HDL | NS |  |
| **C. PCC** |  |  |  |  |  |
| Polyakov 2024,^43^ | Routine regional LND | NS | NS | 8 (0-28) |  |
| Terasaki 2023,^44^ | Regional LND | HDL, CHA, PPH | HDL | LNM-: 14  LNM+: 17 |  |
| Liu ZP 2022,^45^ | Regional LND without LNM evidence on imaging | NS | NS | 4 (1-20) |  |
| Lurje 2019,^34^ | Routine LND | HDL, CHA, PPH, celiac axis | NS | NS |  |
| Ma 2019,^46^ | Regional LND en bloc | HDL, CHA, PPH | HDL | 7 (4-24) |  |
| Kimura 2017, ^47^ | Routine regional LND en bloc | HDL, CHA, PPH | NS | 9 (0-58) |  |
| **Table 2.** continued |  |  |  |  |  |
| Author, publication year | Definition LND | LND location | Frequent LNM region | *LN total |  |
| Giuliante 2016,^48^ | Regional LND | HDL, CHA, PPH, celiac axis | NS | 7 (1-25) |  |
| Mantel 2015,^49^ | Regional systematic LND | HDL, CHA | NS | 4 (1-16) |  |
| Regimbeau 2014,^50^ | LND in all patients | HDL, CHA, portal vein LNs | NS | 5 (1-34) |  |
| Furusawa 2014,^51^ | Standard LND | HDL, CHA, PPH | NS | NS |  |
| Noji 2012,^52^ | Regional LND | HDL, CHA, PPH | NS | NS |  |
| Matsuo 2012,^53^ | Standard sub-hilar LND | Porta hepatis, from CHA to retro-duodenal and hilar LNs | HDL | NS |  |
| Li H 2011,^54^ | Porta hepatis LND en bloc | HDL, CHA, PPH | NS | NS |  |
| Murakami 2011,^55^ | Regional LND | HDL, CHA, PPH | NS | 16 (1-64) |  |
| Chen XP 2009,^56^ | Routine porta hepatis LND | HDL, PPH, celiac axis | NS | NS |  |
| Hasegawa 2007,^57^ | Radical resection with regional LND | HDL, CHA, PPH | NS | NS |  |
| Lai 2005,^58^ | Porta hepatis LND | HDL | NS | NS |  |
| Stein 2005,^59^ | Sub-hilar LND | NS | NS | NS |  |
| Rea 2004,^60^ | Regional LND | HDL, CHA, PPH | NS | NS |  |
| Seyama 2003,^61^ | LND en bloc | HDL, CHA, PPH, celiac axis | NS | NS |  |
| Kitagawa 2001,^62^ | Systematic extended LND | HDL, CHA, PPH, LRV, RRA, para-aortic region | HDL | 24^†^ |  |
