# Supplementary material for: Routine Lymph Node Dissection in the Surgical Treatment of Primary Liver Tumors: a Systematic Review and Meta-Analysis
Source: J Gastrointest Cancer. 2026 Jul 16;57(1):154. doi: 10.1007/s12029-026-01516-9 (PMC13375768; doi:10.1007/s12029-026-01516-9)
Supplement: Supplementary file 7 — Supplementary Table 2 [file 12029_2026_1516_MOESM7_ESM.docx]

**Supplementary Table 2. Random-effects meta-regression of study-level factors.**

| **Group** | **Factor** | **Beta** | **95% CI** | **p-value** | **R² (%)** |
| --- | --- | --- | --- | --- | --- |
| **ICC incidence (n=17)** | Region of study (Asian vs Western) | +0.01 | -0.43 to +0.46 | 0.95 | 0 |
|  | Year of publication | -0.01 | -0.02 to +0.01 | 0.42 | 0 |
|  | Risk of bias (Serious vs Moderate) | +0.06 | -0.19 to +0.31 | 0.64 | 1 |
|  | Sample size (log-transformed) | -0.13 | -0.28 to +0.02 | 0.10 | 0 |
| **PCC incidence (n=21)** | Region of study (Asian vs Western) | +0.09 | -0.07 to +0.26 | 0.27 | 0 |
|  | Year of publication | +0.00 | -0.01 to +0.02 | 0.63 | 0 |
|  | Risk of bias (Serious vs Moderate) | -0.06 | -0.23 to +0.10 | 0.45 | 0 |
|  | Sample size (log-transformed) | +0.05 | -0.09 to +0.19 | 0.51 | 0 |
| **ICC OS HR (n=9)** | Region of study (Asian vs Western) | +0.11 | -0.79 to +1.00 | 0.81 | 0 |
|  | Year of publication | -0.02 | -0.07 to +0.04 | 0.55 | 5 |
|  | Risk of bias (Serious vs Moderate) | -0.35 | -1.11 to +0.40 | 0.35 | 12 |
|  | Tierney-reconstructed HR (yes vs no) | -0.29 | -1.16 to +0.59 | 0.52 | 0 |
| **PCC OS HR (n=17)** | Region of study (Asian vs Western) | -0.23 | -0.55 to +0.09 | 0.15 | 21 |
|  | Year of publication | +0.00 | -0.02 to +0.03 | 0.81 | 0 |
|  | Risk of bias (Serious vs Moderate) | -0.12 | -0.47 to +0.22 | 0.47 | 0 |
|  | Tierney-reconstructed HR (yes vs no) | -0.30 | -0.74 to +0.14 | 0.19 | 0 |

Random-effects meta-regression. Outcome: logit-transformed proportion of LNM (incidence pools) or log hazard ratio (HR pools). Year of publication mean-centered. Sample size log-transformed. Tierney-reconstructed = HR derived from published Kaplan-Meier curve via the method of Tierney et al., R-squared = % of between-study variance explained by the factor
